# Clara cell protein in nasal lavage fluid and nasal nitric oxide - biomarkers with anti-inflammatory properties in allergic rhinitis

**DOI:** 10.1186/1476-7961-10-4

**Published:** 2012-02-06

**Authors:** Kristina Irander, Jörgen P Palm, Magnus P Borres, Bijar Ghafouri

**Affiliations:** 1 Allergy Center, University Hospital, Linköping, Sweden; 2 Department of Physiology and Pharmacology, Karolinska Institutet, Sweden; 3 Department of Pediatrics, Sahlgrenska Academy of Göteborg University, Göteborg, Sweden; 4 Phadia AB, Uppsala, Sweden; 5 Division of Rehabilitation Medicine, Department of Clinical and Experimental Medicine, Faculty of Health Sciences, Linköping University, and Pain and Rehabilitation Centre, County Council of Östergötland, Linköping, Sweden; 6 Occupational and Environmental Medicine, Department of Clinical and Experimental Medicine, Faculty of Health Sciences, Linköping University, and Centre of Occupational and Environmental Medicine, County Council of Östergötland, Linköping, Sweden

**Keywords:** CC16, nasal nitric oxide, allergic rhinitis, anti-inflammatory effects, metachromatic cells, mast cells, basophils, eosinophils, nasal lavage fluid, upper airways

## Abstract

**Background:**

Clara cell protein (CC16) is ascribed a protective and anti-inflammatory role in airway inflammation. Lower levels have been observed in asthmatic subjects as well as in subjects with intermittent allergic rhinitis than in healthy controls. Nasal nitric oxide (nNO) is present in high concentrations in the upper airways, and considered a biomarker with beneficial effects, due to inhibition of bacteria and viruses along with stimulation of ciliary motility. The aim of this study was to evaluate the presumed anti-inflammatory effects of nasal CC16 and nNO in subjects with allergic rhinitis.

**Methods:**

The levels of CC16 in nasal lavage fluids, achieved from subjects with persistent allergic rhinitis (n = 13), intermittent allergic rhinitis in an allergen free interval (n = 5) and healthy controls (n = 7), were analyzed by Western blot. The levels of nNO were measured by the subtraction method using NIOX^®^. The occurrences of effector cells in allergic inflammation, i.e. metachromatic cells (MC, mast cells and basophiles) and eosinophils (Eos) were analyzed by light microscopy in samples achieved by nasal brushing.

**Results:**

The levels of CC16 correlated with nNO levels (r^2 ^= 0.37; p = 0.02) in allergic subjects.

The levels of both biomarkers showed inverse relationships with MC occurrence, as higher levels of CC16 (p = 0.03) and nNO (p = 0.05) were found in allergic subjects with no demonstrable MC compared to the levels in subjects with demonstrable MC. Similar relationships, but not reaching significance, were observed between the CC16 and nNO levels and Eos occurrence. The levels of CC16 and nNO did not differ between the allergic and the control groups.

**Conclusions:**

The correlation between nasal CC16 and nNO levels in patients with allergic rhinitis, along with an inverse relationship between their levels and the occurrences of MC in allergic inflammation, may indicate that both biomarkers have anti-inflammatory effects by suppression of cell recruitment. The mechanisms behind these observations warrant further analyses.

## Background

Clara cell protein (CC16, identical to CC10 and uteroglobin) is a biomarker of high interest in airway diseases. The protein, initially described in the epithelium of the tracheobronchial tree as a secretory product from non-ciliated Clara cells, diffuses passively from the respiratory tract into serum and is excreted via the urinary tract [1]. CC16 is ascribed a protective role against oxidative stress and inflammation in the respiratory tract [2]. Due to a particular vulnerability of Clara cells to lung toxicants CC16 has also been evaluated as a useful biomarker of respiratory epithelial damage in acute and chronic exposures to airway irritants [1-3]. Most studies have focused on the lower airways with results based on CC16 levels in serum, sputum and bronchoalveolar lavage fluid. In asthmatic children and adults lower levels have been found compared to healthy controls [3].

Although CC16 also has been demonstrated in nasal lavage fluid [NLF] [4], only a few studies on nasal levels have been reported. Thus, the CC16 levels in NLF related to exposures to air pollutions have been analyzed, with decreased levels found in a group of epoxy workers with chronic exposure to an irritating chemical [5], in contrast to increased levels after acute exposure to hot humid ozone-polluted environment in combination with physical exercise [6]. In intermittent allergic rhinitis due to pollen allergy the levels were lower in patients compared to controls during the pollen season [7,8], and an inverse relation between nasal CC16 levels and symptoms and signs of rhinitis were observed after allergen-challenge [9]. No study analyzing the nasal CC16 levels in persistent allergic rhinitis has up to now been found.

The aims of this report were, besides analyses of nasal CC16 levels in subjects with persistent allergic rhinitis and subjects with intermittent allergic rhinitis during a symptom-free interval, to evaluate the presumed protective role of nasal CC16 in allergic inflammation. The CC16 levels were therefore related to nasal nitric oxide (nNO) that is present in high concentrations in the upper airways, and considered a biomarker with beneficial effects, due to inhibition of bacteria and viruses along with stimulation of ciliary motility [10]. Furthermore, the analyses included the relations between the levels of both CC16 and nNO and the occurrence of nasal metachromatic cells (MC, mast cells and basophils) and eosinophils (Eos), i.e. the major effector cells in IgE-mediated allergic inflammation.

## Methods

### Subjects

The subjects were included in a cohort during infancy and followed regarding development of allergy symptoms [11-13]. At the 18-year follow-up we took the opportunity to analyze the expression of different biomarkers in the upper airways. The examinations were performed out of pollen season and the subjects had to be free from airway infections for at least 10 days prior to the examination, thus with all subjects being in a period in optimal good health. The diagnoses of upper and lower airway allergy and atopic dermatitis were based on clinical histories of allergy symptoms and careful clinical examinations as previously described in detail [13].

Allergic rhinitis subjects (n = 18), with or without bronchial or skin symptoms were included in this report, while individuals suffering from dermatitis without airway problems or presenting inconclusive symptoms were excluded. Subjects reporting use of nasal decongestants, local or systemic steroids or smoking habits, factors with known or suspected effects on the CC16 and nNO levels, were too low in number to be evaluated separately and therefore not included. All of the rhinitis subjects were sensitized to one or more allergens according to a skin prick test using ALK extracts (ALK, Sweden AB) with perennial allergens (horse, cat, dog, D. pteronyssinus, D. farinae, Alternaria, Cladosporium) and pollen allergens (birch, timothy, mugwort). The rhinitis subjects were separated into a persistent allergy sub-group (n = 13) due to sensitization to perennial allergens regardless of any concurring positive pollen tests, and an intermittent allergy sub-group (n = 5), sensitized to pollens only and thus not exposed to any offending allergens for at least three months. Healthy, non-sensitized subjects served as a control group (n = 7).

Current symptoms on the day of examination, including four rhinitis symptoms (itching, sneezing, secretion and obstruction) and four bronchial symptoms (cough, mucous production, wheezing and dyspnea) were scored from 0 (no symptoms) to 10 (disabling symptoms) on visual analogue scales.

### Nasal lavage samples

Nasal lavage was performed using saline pre-warmed to 37°C. The subject kept the head bent forward with the face held horizontally, while the left nasal cavity was filled with saline, using a 10 ml syringe connected to the nostril via a short tube and a nasal olive. After five minutes approximately five ml of the saline could be recovered by aspiration. The samples were centrifuged to remove cellular debris and aliquots of the supernatants were stored at - 20°C in eppendorff tubes until analysis. Total protein concentrations were determined with Bio-Rad protein assays according to Bradford [14].

### Western blot analysis of CC16 levels

Proteins from NLF were separated using SDS-PAGE, with a gradient gel range T: 5-20% and C: 1.5% and a stacking gel with T: 5% and C: 5% on Mini-Protean II electrophoresis cell from Bio-Rad Laboratories. Samples, 40 μg of protein, were mixed 1:1 with cocktail (10% w/v SDS, 150 mM DTT, 1% w/v bromphenol blue, 0.5 mM Tris-HCl pH 6.8, glycerol). The samples were boiled for three minutes, according to standard procedures, before loaded in the wells on the SDS-PAGE and run in electrode buffer (0.16% w/v Tris, 0.72% w/v glycine, 0.05% w/v SDS). The SDS-PAGE was run for approximately 30 minutes in 100 V, 60 mA and then elevated to 200 V until finished.

SDS-PAGE gels were blotted on Immun-Blot PVDF Membrane using Mini Trans-Blot Electrophoretic Transfer Cell (Bio-Rad Laboratories). Membranes were blocked in Tris-buffered saline (40 mM Tris-HCl, 500 mM NaCl, pH 7.5) with 5% non-fat dried milk over night. Membranes were washed with Tween-20 Tris-buffered saline (TTBS: 40 mM Tris-HCl, 500 mM NaCl, 0.05% Tween-20) and incubated with primary antibody against CC16 (Biovendor RD181022220) in TTBS with 2% non-fat dried milk over night. The membranes were washed with TTBS and followed by incubation with HRP-conjugated secondary antibody (anti-goat/sheep IgG, SIGMA, MI, USA) for 1 h. The latter wash procedure was repeated once pursued by detection of antigen/antibody conjugate with ECL (GE Healthcare) and developed on X-ray film. The X-ray films were analyzed as digitized images using a CCD (charged-coupled device) camera (1340 × 1040 pixels) in combination with a computerized imaging 12-bit system. The amount of protein in a band was assessed as optical density (OD).

### Measurements of nitric oxide from the upper and lower airways

Measurements of nitric oxide (NO) followed the ATS recommendations [15] using NIOX^® ^(Aerocrine AB, Sweden) at a flow rate of 3 l/min. The values of NO from the upper airways (nNO) were calculated according to the subtraction method [16], regarded to be the best validated method where a transnasal flow is used [15]. Briefly, when measuring nasally exhaled NO, a tight-fitting nasal mask was adapted to the mouthpiece used for oral exhalations, and the mean of three recordings was calculated. Before measuring the orally exhaled NO (eNO), the subject performed a mouthwash with 20 ml of sodium bicarbonate solution (10%) for 1 minute, in order to avoid the non-airway contribution of NO from saliva in the oropharyngeal tract [17]. The mean from three immediately performed recordings was calculated. This eNO mean value was subtracted from the mean value of nasally exhaled NO, in the calculation of nNO. Thus, nNO as calculated using the subtraction method represents the supra-velar airway contribution to nasally exhaled NO.

### Cytospin preparations of nasal mucosal cells

Mucosal cells were harvested from the right nasal cavity by a gentle nasal brushing using a 5.5 mm diameter nylon brush (Doft AB, Östhammar, Sweden). The brush was immediately placed in a tube containing physiological saline and twirled for 3-5 seconds. After cytocentrifugation onto glass slides, the materials were air-dried and fixated in 95% ethanol or methanol for later staining with toluidine blue for visualization of MC and Wright's stain for visualization of Eos, respectively. Blinded analysis of the cells on coded slides was performed by light microscopy (magnification × 250). Occurrence of one or more of the cells was regarded as a positive result (MC^pos ^and Eos^pos^, respectively), and absence of cells as a negative result (MC^neg ^and Eos^neg^, respectively), provided the density of epithelial cells were > 25 cells per visual field.

### Statistical analyses

In general, statistics were analyzed by non-parametric methods, using the Graph Pad Prism software program. Mann-Whitney U Test was used in calculations of differences between two groups. Results are presented as mean values ± 1 standard deviation. Spearman Rank Correlation was used in evaluating correlations. A p-value of < 0.05 was regarded as significant.

### Ethics

The study was performed according to the Helsinki Declaration and approved by the Ethical committee at the University Hospital in Linköping, Sweden (03694). A written informed consent was obtained from each of the participants.

## Results

### Symptom scores

The combined scores of the four nasal symptoms and the four bronchial symptoms are presented in Table 1. The symptoms were mild with only slightly higher score values in the persistent allergy sub-group with current allergen exposure.

**Table 1:** Results of the analyzed parameters in the allergy sub-groups and the control group

	Allergy sub-groups		Control	Group
	**persistent**^**a**^ (n = 13)	**intermittent**^**b**^ (n = 5)	**group**^**c**^ (n = 7)	differences
Combined scores of: rhinitis symptoms	2.3 ± 2.8	0.7 ± 1.5	0.4 ± 1.1	a/c p = 0.06
bronchial symptoms	0.6 ± 1.3	0.0	0.0	n.s.

Levels of:				
CC16 (OD)	15 ± 9	18 ± 12	11 ± 9	n.s.
nNO (ppb)	70 ± 34	63 ± 30	79 ± 33	n.s.
eNO (ppb)	38 ± 41	13 ± 8	13 ± 5	a/b p = 0.06 a/c p = 0.05

Number of subjects:				
MC^pos ^/MC^neg^	7/6	2/3	0/7	(a+b)/c p = 0.03
Eos^pos ^/Eos^neg^	9/4	2/3	2/4	n.s.

Symptom scores, levels of CC16, nNO and eNO and the number of subjects with/without demonstrable metachromatic cells (MC^pos^/MC^neg^) and eosinophils (Eos^pos^/Eos^neg^) in the allergy sub-groups and the control group. OD: optical density.

### Protein concentrations in nasal lavages

The concentrations of the total amount of proteins in NLF were 350 ± 340 μg/ml (persistent allergy sub-group), 440 ± 260 μg/ml (intermittent allergy sub-group) and 320 ± 310 μg/ml (control group). No significant statistical differences between the groups were found.

### Levels of CC16, nNO and eNO and occurrences of MC and Eos in relation to allergic rhinitis

**Figure 1:**
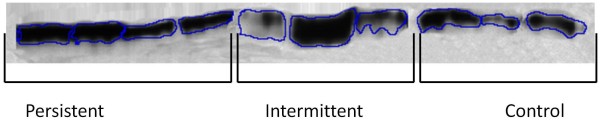
**A typical western blot of expression level of CC16 in nasal lavage fluid from 4 subjects with persistent allergic rhinitis, 3 subjects with intermittent allergic rhinitis and 3 controls**. 40 μg total proteins were analyzed from each subjects.

The levels of CC16 were slightly, but not significantly, higher in the allergy sub-groups compared to the control group (Table 1, Figure 1).

The nNO levels were slightly lower in the two allergy sub-groups compared to the control group, but with no significant differences. In contrast, eNO was higher in the sub-group with persistent allergy compared to the sub-group with intermittent allergy (p = 0.06) and to the control group (p < 0.05) (Table 1).

Analyses of cell occurrences showed positive results (MC^pos ^and Eos^pos^) in subjects from the two allergy sub-groups, but some of the members in both sub-groups had negative results (MC^neg ^and Eos^neg^). In the control group MCs were not found in any subjects, whereas Eos were found in two of them (one slide was failed) (Table 1).

Thus, the sub-group with persistent rhinitis was discerned from the sub-group with intermittent rhinitis and the healthy control group only by eNO, but not by any of the upper airway markers.

### Correlation between CC16 and nNO levels

**Figure 2:**
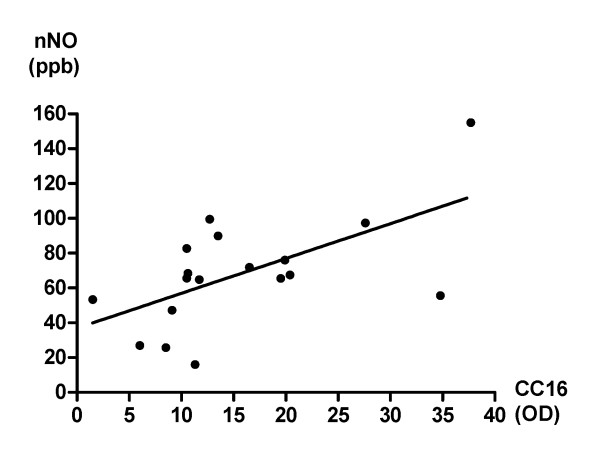
**Correlation between CC16 and nNO levels**. Correlation between the CC16 and nNO levels in subjects with allergic rhinitis (n = 18; r^2 ^= 0.37; p = 0.02).

A significant positive correlation was found between the CC16 and the nNO levels in the combined allergic rhinitis sub-group (n = 18; r^2 ^= 0.37; p = 0.02; Figure 2).

### Levels of CC16 and nNO in relation to occurrences of MC and Eos

**Figure 3:**
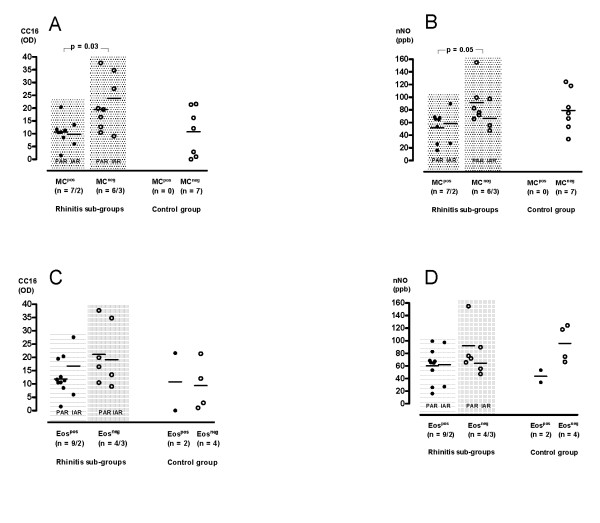
**Levels of CC16 and nNO in relation to occurrences of effector cells in allergic inflammation**. Levels of CC16 and nNO in the two groups of subjects with allergic rhinitis and the control group in relation to the occurrence of metachromatic cells (A, B) and eosinophils (C, D). PAR: persistent allergic rhinitis; IAR: intermittent allergic rhinitis. MC^pos ^and MC^neg^: subjects with and without demonstrable metachromatic cells; Eos^pos ^and Eos^neg^: subjects with and without demonstrable eosinophils.

The levels of both CC16 and nNO in the combined sub-group of allergic rhinitis were significantly higher in subjects with MC^neg ^results compared to the levels in subjects with MC^pos ^results (21 ± 19 OD vs 10 ± 5 OD; p = 0.03 and 83 ± 32 ppb vs 53 ± 25 ppb; p = 0.05, respectively; Figure 3A-B).

In subjects with Eos^neg ^higher levels of CC16 (20 ± 12 OD vs 13 ± 7 OD) and nNO (80 ± 36 ppb vs 61 ± 28 ppb) were found compared to the levels in subjects with demonstrated Eos, although these differences did not reach significance (Figure 3C-D).

Thus, inverse relationships were found between both CC16 and nNO levels and MC occurrence with the same tendency of inverse relationships and Eos occurrence.

## Discussion

In this study, we observed new relations between nasal CC16, nNO and MC (one of the effector cells in allergic inflammation). The levels of CC16 and nNO correlated significantly. The levels of both CC16 and nNO correlated inversely to the occurrence of MC, but the differences between the lower levels of the two biomarkers in subjects with demonstrable Eos compared to the levels in subjects with no demonstrable Eos did not reach significance.

Our results of CC16 levels in relation to MC and Eos are in accordance with results in studies of the lower airways. Thus, an inverse correlation was found between Clara cell numbers and mast cell numbers in the small airways [18], and a negative correlation between the CC16 levels and Eos numbers in induced sputum was found in atopic asthmatics [19].

However, our result of nNO levels and the occurrence of Eos in the nasal mucosa is not in accordance with results in asthma studies, where eNO is supposed to reflect eosinophilic inflammation [20-22]. Relations between eNO levels and MC findings in the lower airways have to our knowledge not been analyzed.

We have no unequivocal explanations to our observations. Undoubtedly, inflammatory events in allergic inflammation are very complex. However, the quality of airway protection of both CC16 and nNO as reported in previous studies is of interest. Thus, CC16 is regarded to have anti-inflammatory effects by inhibition of PLA_2 _activity on arachidonic acid, reducing the availability of the pro-inflammatory agents such as prostaglandins and leukotriens and the chemotaxis of various inflammatory cells [2]. It is suggested that nNO is involved in many processes with regulatory, protective, defensive and also deleterious effects [23]. The protective function, however, is regarded to be of importance, as the high concentrations in the upper airways are supposed to have a direct inhibitory effect on the growth of pathogens and thus enhance the local host defense against microbial infections [10]. Regarding the lower airways, orally exhaled NO is considered a biomarker of inflammation, particularly in allergic asthma [20]. Interestingly, two recent *in vitro *studies of the effect of NO donors and NO synthase inhibitors on antigen induced contractile responses and pro-inflammatory mediator release in peripheral lung tissue, support the belief that NO has a protective anti-inflammatory effect also in lung parenchyma [24,25]. In a review of *in vivo *and *in vitro *studies of NO and the regulation of mast cell activation, the conclusion was that the protective anti-inflammatory role of NO was predominant over pro-inflammatory effects, due to the inhibitory actions of NO on mast cell degranulation with decreased mediator release and expression of cytokines [26].

Our observations are in accordance with the concept of the anti-inflammatory roles of CC16 and nNO, as the inverse relationships between the levels of the biomarker and the occurrence of the effector cells might be explained by suppressive effects on recruitment of these cells. However, no analyses of mediators or cytokines related to MC or Eos recruitment were included in our study, why further studies are necessary to clarify the mechanisms behind the observations. Likewise, the correlation between the levels of CC16 and nNO has to our knowledge not been reported before, and this observation also needs further analyses to be explained.

In the analyses of CC16 and nNO levels in the allergy sub-groups and the control group no differences were found between the groups. This failure of CC16 to discern allergic subjects is in disagreement with the results from the other studies of CC16 in NLF [7-9], where significantly lower CC16 levels were found in subjects with current intermittent allergic rhinitis compared to healthy controls. The different outcomes might be due to a more modest exposure to perennial allergens compared to allergen exposures during pollen seasons, a notion supported by the low score values of nasal symptoms in our participants. The different results might also, at least in part, be explained by the methods used for analysis of the CC16 levels, which was Western blot in our study and ELISA in the studies referred to above [7-9].

The failure of nNO to discern allergic rhinitis groups from controls is, however, in agreement with other studies [27-29] including the one using the same subtraction method for nNO calculation [30]. In contrast, measurement of eNO levels is considered to be a useful tool in diagnosis and management of asthma [20,21]. Interestingly, eNO measurements in our study discerned the persistent allergy group with current allergen exposure, even though very low bronchial symptom scores were reported.

A drawback of our study is the low numbers of participants, which was due to the use of a cohort designed for longitudinal follow-ups not permitting substitution of subjects excluded for various reasons. Furthermore, the impact of CC16 and nNO on rhinitis symptoms could not be evaluated, as the score values were too low for permitting any tenable conclusion. Although we regard our results as pilot observations, we consider them to be relevant, as all analyses were performed according to validated methods.

In order to avoid impact on nNO results due to a reduced contribution from the paranasal sinuses we found it to be an advantage to perform the examinations of the subjects being in an optimal health condition with no airway infections. No subject suffered from sinusitis symptoms. Congestion of the nasal mucosa was observed in three individuals with persistent allergy, but their nNO values were above or slightly below to the mean value of the sub-group. Thus, occlusion of the sinus ostiae was not suspected in any of the subjects.

## Conclusions

Although CC16 in NLF and nNO were found not to be applicable biomarkers of current allergy in groups of subjects with very mild rhinitis symptoms or in groups during a free interval of allergen exposure, new details have been observed supporting the concept of airway protection in allergic rhinitis. Thus, the correlation between nasal CC16 and nNO levels and the inverse relationships between these biomarkers and the occurrence of MC in allergic rhinitis are results, which may indicate protective properties of both CC16 and nNO by suppression of cell recruitment in upper airway inflammation.

As the results are pilot observations, further studies are needed to clarify the mechanisms behind the observations and to analyze the impact of the two biomarkers on rhinitis symptoms.

## List of abbreviations

NLF: nasal lavage fluid; NO: nitric oxide; nNO: nasal nitric oxide; eNO: exhaled nitric oxide; CC16: clara cell protein 16; MC: metachromatic cells; i.e. mast cells and basophils; Eos: eosinophils

## Competing interests

The authors declare that they have no competing interests.

## Authors' contributions

KI, JPP and MPB designed the study. KI and BG designed the analysis of CC16. BG carried out the CC16 analyses. KI and BG evaluated the results and KI performed the statistical analyses. KI, BG and MPB wrote the manuscript. All authors have read, revised and approved the manuscript.
